# Infectious Agents Associated with Abortion Outbreaks in Italian Pig Farms from 2011 to 2021

**DOI:** 10.3390/vetsci11100496

**Published:** 2024-10-12

**Authors:** Anna Donneschi, Matteo Recchia, Claudia Romeo, Paolo Pozzi, Cristian Salogni, Antonio Marco Maisano, Giovanni Santucci, Federico Scali, Silvia Faccini, Maria Beatrice Boniotti, Mario D’Incau, Dominiek Maes, Giovanni Loris Alborali

**Affiliations:** 1 Istituto Zooprofilattico Sperimentale della Lombardia e dell’Emilia Romagna—IZSLER, 25124 Brescia, Italymatteo.recchia@izsler.it (M.R.); cristian.salogni@izsler.it (C.S.); antoniomarco.maisano@izsler.it (A.M.M.); giovanni.santucci@izsler.it (G.S.); federico.scali@izsler.it (F.S.); silvia.faccini@izsler.it (S.F.); mariabeatrice.boniotti@izsler.it (M.B.B.); mario.dincau@izsler.it (M.D.); giovanni.alborali@izsler.it (G.L.A.); 2 Center for Evolutionary Hologenomics—Globe Institute, University of Copenhagen, 1350 Copenhagen, Denmark; 3 Dipartimento di Scienze Veterinarie, Università degli Studi di Torino, 10095 Grugliasco, Italy; paologiuseppecarlo.pozzi@unito.it; 4 Department of Internal Medicine, Reproduction and Population Medicine, Faculty of Veterinary Medicine, Ghent University, 9820 Merelbeke, Belgium; dominiek.maes@ugent.be

**Keywords:** abortion-inducing pathogens, diagnostic protocol, farrowing unit, porcine fetuses, reproductive failure, sow

## Abstract

**Simple Summary:**

Abortions are a significant contributor to economic losses in the swine breeding industry. Identifying the factors responsible for abortion outbreaks is crucial for optimizing farm management and implementing preventive measures, though this can be challenging due to their often multifactorial nature. In this study, we retrospectively examined the infectious agents associated with abortion outbreaks from 2011 to 2021 in northern Italy. The most frequently detected pathogens in fetal samples were porcine reproductive and respiratory syndrome virus (PRRSV), porcine circovirus-3 (PCV3), and PCV2, while *Chlamydia* spp., porcine parvovirus (PPV), and *Leptospira* spp. were less common. PRRSV prevalence fluctuated yearly without a clear trend, whereas PCV2 showed a slight decline and PCV3 increased over the study period. Our findings suggest a general decrease in abortion outbreaks between 2011 and 2021. PRRSV, PCV2, and PCV3 were commonly detected in aborted fetuses, while pathogens like *Chlamydia* spp. and *Leptospira* spp. had a limited impact, possibly due to improved on-farm hygiene and biosecurity measures.

**Abstract:**

The present study retrospectively analyzed the infectious agents associated with 829 abortion outbreaks occurring from 2011 to 2021 in northern Italy. Foetuses were subjected to necropsies, and organ samples were analyzed by direct PCR to screen for six swine pathogens. In 42.0% of the examined outbreaks, at least one infectious agent was found. Porcine reproductive and respiratory syndrome virus (PRRSV) (24.9%) and porcine circovirus-2 (PCV2) (11.5%) were the most frequently detected among the known abortion-inducing pathogens. *Chlamydia* spp. (5.6%), porcine parvovirus (PPV) (4.0%), and *Leptospira* spp. (2.6%) were less common. Although its role in swine reproductive disorders is still unclear, PCV3 was detected in 19.6% of the cases. Coinfections were detected in 25.0% of positive outbreaks, and the most frequent coinfection was represented by PRRSV and PCV2 (32.2%), followed by PRRSV and PCV3 (23%). PCV2 prevalence showed a slight but consistent reduction during the study period, while PCV3 increased in frequency. Our data suggest an overall reduction in abortion outbreaks during the study period. PRRSV was confirmed as the main abortion agent detected in the examined area, while PCV2 prevalence showed a decline. Conversely, PCV3 detection has been increasing, supporting its potential role as an abortion agent. Our results highlight the importance of implementing a consistent and standardized sampling procedure, as well as a thorough diagnostic protocol, to reduce the incidence of inconclusive diagnoses.

## 1. Introduction

Reproductive failure in pig production results from a series of dynamic conditions that significantly affect breeding herds’ profitability. The clinical presentation of reproductive disease varies greatly depending on the etiology and stage of gestation and includes embryonic death and resorption, abortion, mummification, stillbirth, and weak-born piglets with increased neonatal mortality [1,2]. Abortion (i.e., the expulsion of dead fetuses between days 35 and 109 of gestation) constitutes a major cause of economic losses, especially when it occurs at the end of pregnancy. Abortion rates within the threshold of 2% of pregnant sows per year are considered acceptable in the swine industry [3], but when this value is exceeded, the consequences on reproductive parameters become relevant [4]. The physiology of pregnancy can be disrupted by both infectious agents and non-infectious factors [5]. Non-infectious causes are estimated to account for 70% or more of fetal deaths [2] and include, among others, environmental parameters, management practices [6], diet, and toxic substances [7]. Infectious abortions can be related to the systemic effect of acute maternal diseases or to local infections of one or more fetal–placental units.

Viruses are considered the major infectious cause of pregnancy/reproductive failure in swine, and the most important are porcine reproductive and respiratory syndrome virus (PRRSV), porcine circovirus-2 (PCV2), porcine parvovirus (PPV), pseudorabies virus (PRV), encephalomyocarditis virus (EMCV), and classical swine fever virus (CSFV) [5]. PRRSV is probably the most relevant abortion-inducing pathogen in the swine industry and causes severe economic losses in breeding farms due to late-term abortions, premature farrowing, and increased piglet mortality [8]. Increased pre-weaning mortality from 4 to 17% and a decreased farrowing rate have also been reported during PRRS outbreaks in different countries [9]. The severity of the reproductive failure induced by PRRSV will depend on the viral strain, pregnancy stage, sow genetics and immune status, herd management, and the general health status of the farm [8,10]. PCV2-associated reproductive failure is characterized by the occurrence of late abortions, stillbirths, and fetal cardiac lesions characterized by extensive fibrosis and/or necrotizing myocarditis, with high amounts of PCV2 in myocardial lesions and other fetal tissues [11]. Finally, PPV is a well-known cause of reproductive disorders in sows, leading to stillbirth, mummification, embryonic death, and infertility [12].

In addition to the viral pathogens mentioned above, which are well-known abortion agents, porcine circovirus-3 (PCV3) has been recently speculated to play a role in reproductive diseases of sows [13]. PCV3 was identified as a novel virus in 2016, in association with cases of cardiac and multiorgan inflammation [14], porcine dermatitis and nephropathy syndrome (PDNS), and reproductive failure [15]. The vertical transmission of PCV3 is supported by its high placental tropism, but field data suggest that the occurrence of reproductive failure is strongly dependent on the strain and viral load observed in sows [16,17].

Primary bacterial agents associated with swine abortion include *Chlamydia* spp., *Leptospira* spp., *Brucella suis*, and *Erysipelothrix rhusiopathiae* [2,5]. *Chlamydiaceae* infection in pig herds is often subclinical but has been variously associated with respiratory signs, pericarditis, enteritis, conjunctivitis, and reproductive disorders in both males and females [18]. *Leptospira* spp. is a zoonotic bacterium of worldwide relevance, for which pigs, like many other mammals, can act as maintenance hosts. The infection in swine is known to cause fetal disease and genital lesions [19,20]. Other bacteria potentially related to abortions, such as *Escherichia coli*, *Streptococcus* spp., and *Staphylococcus* spp., are considered opportunistic agents, which mainly reach the fetuses retrogradely from the open cervix or due to bacteremia [21].

One of the main challenges in the management of reproductive disorders in pig production concerns their multifactorial nature, and it is not uncommon to find microbial coinfections within the same abortion outbreak [22]. Therefore, a thorough knowledge of abortion outbreaks’ etiology and associated epidemiology, as well as their evolution over time, is pivotal for optimizing sow health management and implementing on-farm preventive and control measures. This is particularly important within high-density pig breeding areas such as northern Italy, where infectious agents can be transferred more easily between farms [22]. Epidemiological studies drawing a comprehensive overview of infectious abortigenic agents in Italy are scarce and limited to short periods of time [22].

Based on these considerations, we retrospectively investigated the infectious etiology of abortion outbreaks within a high-density pig breeding area of northern Italy over 11 years, analyzing annual and seasonal trends in pathogens associated with abortions and evaluating potential associations with the density of swine in the area.

## 2. Materials and Methods

### 2.1. Sample Overview

**Figure 1:**
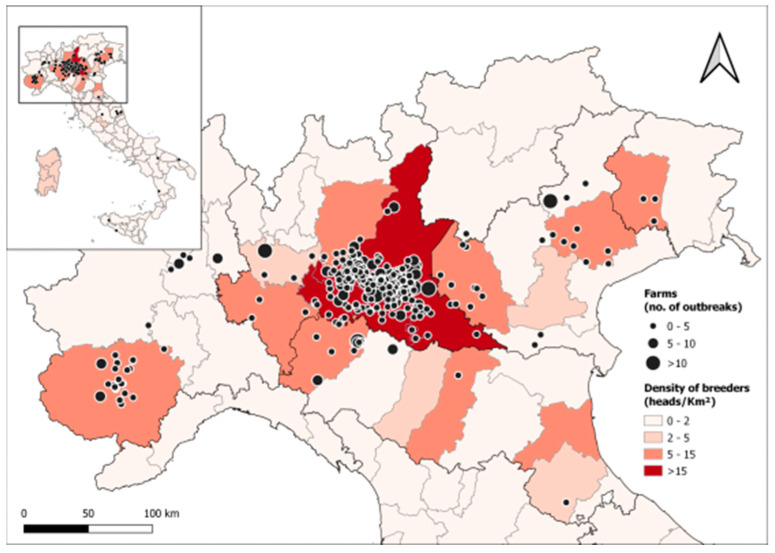
Location of Italian swine breeding farms that submitted aborted fetuses to the Istituto Zooprofilattico Sperimentale della Lombardia e dell’Emilia Romagna (IZSLER, Brescia, Italy) for analysis between 2011 and 2021, with a focus on northern Italy, where most of the samples came from. As detailed in the figure legend, the circle size is proportional to the number of abortion outbreaks in each farm during the study period, and background colors indicate the density of breeders in each administrative province.

We retrospectively evaluated all the submissions of aborted pig fetuses received for analysis between 2011 and 2021 by the Istituto Zooprofilattico Sperimentale della Lombardia e dell’Emilia Romagna (IZSLER, Brescia, Italy). Foetuses were submitted by herd veterinarians to IZSLER for reproductive disease monitoring in case of abortion events on the farm. The dataset spans 11 years and includes 1224 submissions from 293 different breeding farms, corresponding to a total of 5474 fetuses. Samples were submitted by farms located in 15 out of the 22 Italian administrative regions, with a preponderance of submissions (97% of samples) from the four North Italian regions (Lombardy, Piedmont, Veneto, Emilia Romagna), where the vast majority of breeders (73%) are located (Figure 1).

The density of swine in these four regions was obtained from the Italian Veterinary Registry (URL: www.vetinfo.it, accessed on 19 December 2022) and was on average 83.1 heads/Km^2^ and 5.2 breeders/Km^2^, ranging from a minimum of 0.158 heads and 0.013 breeders per Km^2^ (VB province, Piedmont) to a maximum of 531 heads and 32.2 breeders per Km^2^ (CR province, Lombardy).

The total number of submissions per year ranged from 71 to 135 with an average of 4 fetuses/submission (1–25), and the number of submitting farms per year ranged from 41 to 64. Most of the farms (60.1%) submitted samples only in one year, but 7.2% of them submitted samples over five or more years. Different submissions from the same farm submitted within three months from the first case were considered a single abortion outbreak and grouped for analysis, resulting in a total of 829 abortion outbreaks over the whole study period.

### 2.2. Sample Processing and Pathogen Detection

Herd veterinarians followed a standardized sampling protocol: IZSLER recommends the collection of at least 3 aborted fetuses per litter, and fetuses from different litters need to be stored separately with a clear indication of the abortion date. Whenever possible, the placenta should be sampled as well. Samples must be collected according to good veterinary practices, stored at 4 °C, and submitted within 24 h from collection. Once received by IZSLER, aborted fetuses were subjected to thorough necropsies [23], and lung, heart, and liver samples were collected from each fetus. Considering fetal length measured from the top of the head to the base of the tail, each case was classified as early (i.e., up to 15 cm) or late (>15 cm) abortion. The different organs were pooled by litter (up to five fetuses per pool), homogenized (10% *w*/*v* in modified Eagle medium), and analyzed for the presence of the following pathogens: PCV2, PCV3, PPV, PRRSV, and *Chlamydia* spp. Kidney samples from each fetus were instead analyzed for the presence of *Leptospira* spp. As placenta samples were only available for a limited number of cases enrolled in this study, these data were not included in our analyses. The diagnostic methods applied to each pathogen are reported in Table 1.

The real-time RT-PCR protocol used for PRRSV detection allowed us to differentiate between viral genotypes 1 and 2. Conversely, the protocol used to investigate PCV2 prevalence did not provide genotype differentiation. Based on current epidemiological knowledge, a different positivity cut-off was considered for PCV2 and PCV3 [13,29,30].

The two bacteria were routinely included in the abortion diagnostic protocol only from 2015 onwards. For PCV3, routine analysis through real-time PCR [15] has been performed at IZSLER only since 2017, but archived samples from 2015 to 2016 were also retrieved and analyzed with this method. Regarding PPV, cases collected between 2011 and 2014 were subjected to molecular testing as described in [25]. The method reported in [26] was adopted by IZSLER in routine diagnostics only from 2021, but for the present study we applied it retrospectively to archived samples from 2015 onwards.

Previously analyzed positive samples, confirmed by Sanger sequencing, were used as positive controls in real-time PCRs for all the qualitative analysis. The cut-offs for positivity are listed in Table 1. For the quantitative real-time PCR for PCV2, a recombinant plasmid containing the synthetic sequence of a portion of the ORF1 of the strain NC_005148.1 (GenBank NC_005148.1 from nt 518 to 917) was purchased from Eurofins Genomics, Ebersberg, Germany. Replicates of tenfold serial dilution in nuclease-free water were used to estimate the viral load by quantitative qPCR. An outbreak was considered positive for a specific agent when at least one sample tested positive for it.

### 2.3. Statistical Analysis

First, we analyzed temporal trends in abortion outbreaks within the study period. Before analysis, the absolute number of outbreaks by year and season was normalized by the total number of swine samples submitted for analysis in each time period to account for any underlying variation in the volume of analyses carried out at IZSLER during the study period. The obtained proportion was then modeled through a beta regression, including season, year, and a quadratic year effect as explanatory variables. Second, variation in the prevalence of specific etiological agents within the outbreaks was analyzed through a set of mixed logistic regressions: presence/absence of each pathogen represented the response variable; year, season, and density of pigs within the farm’s province (no. of heads/km^2^) were included as explanatory variables. Farm ID was always included as a random factor to account for repeated submissions by the same farm. In the case of significant annual and/or seasonal effects, pairwise comparisons between years and seasons were carried out through *t*-tests on differences in least squares means, applying Holm correction for multiple comparisons. Overdispersion was checked through the generalized chi-square/degrees of freedom ratio. All analyses were carried out through PROC GLIMMIX in SAS/STAT 9.4 software (SAS Institute Inc., Cary, NC, USA).

## 3. Results

### 3.1. Temporal Trends in Abortion Outbreaks and Prevalence of Abortion-Inducing Pathogens

**Figure 2:**
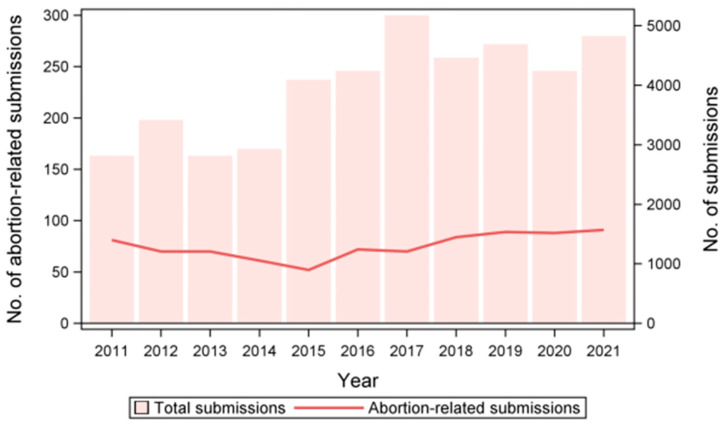
Number of swine samples submitted for analyses to IZSLER and number of abortion-related submissions by year.

We identified a total of 829 abortion outbreaks over the whole study period, with a median value of 1 outbreak/farm (range: 1–25) and a number of outbreaks per year ranging from 52 to 91. Although, in absolute terms, the number of outbreaks appeared to increase during the study period, once corrected for the number of swine sample submissions, we detected a nonlinear decrease in abortion outbreaks (χ^2^_1_ = 18.8; *p* < 0.0001; Figure 2).

Indeed, the total number of swine sample submissions processed at IZSLER increased strongly during the study period, from 2818 in 2011 to 4829 in 2021. Consequently, in 2011, the samples related to abortion outbreaks represented 11.3% of all swine samples submitted to IZSLER, while in 2021 this was only 7.5%, reaching a minimum of 5.1% in 2015. Additionally, in all years, the submissions of aborted fetuses were less frequent in summer than in autumn and winter (both *p* < 0.04).

The total number of outbreaks tested for target pathogens ranged from a minimum of 438 cases tested for PCV3 to a maximum of 817 cases tested for PRRSV. In 348 out of 829 abortion outbreaks (42.0%), at least one of the tested pathogens was detected. The pathogens most frequently detected were PRRSV (24.9%), PCV3 (19.6%), and PCV2 (11.5%), while *Chlamydia* spp. (5.6%), PPV (4.0%), and *Leptospira* spp. (2.6%) were less common. In all cases, PRRSV strains belonged to genotype 1.

Detailed prevalence data for each of the tested pathogens are reported in Table 2. Notably, while only 11.5% of the samples showed PCV2 loads greater than the set positivity threshold (see Table 1), the virus was actually detected (i.e., PCR Cq < 40) in 29.5% of aborted fetuses.

Overall, most of the outbreaks were characterized by late-term abortions (73.5%). PRRSV and *Chlamydia* spp. infections were disproportionally associated with late term (83.1% of PRRSV-positive outbreaks; χ^2^_1_ = 12.1; *p* = 0.0005) and early (59.3% of *Chlamydia*-positive outbreaks; χ^2^_1_ = 20.3; *p* < 0.0001) abortions, respectively.

Multiple agents were detected in 25.0% (87/348) of positive outbreaks. Most of these coinfections (74/87) were associated with two different agents, but in a few cases (13/87), three different pathogens were detected. The most frequent coinfection was represented by PRRSV and PCV2 (28/87), followed by PRRSV and PCV3 (20/87). All the other combinations were found more rarely (see Table 3).

**Figure 3:**
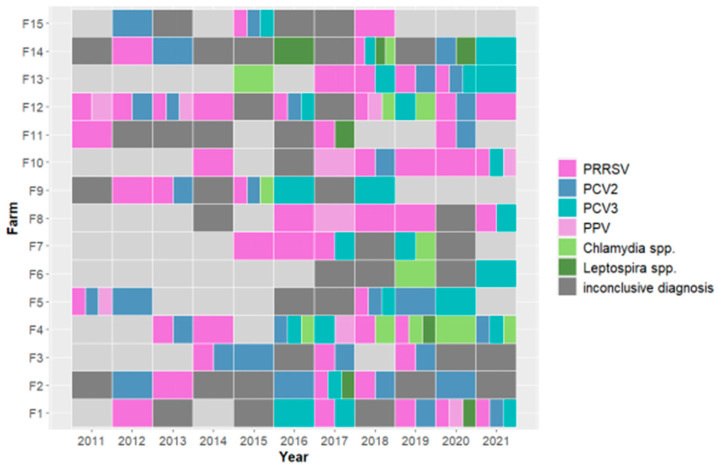
Infectious agents were detected in abortion outbreaks occurring from 2011 to 2021 in the subset of 15 pig farms with the highest number of outbreaks over the period. PCV3, *Chlamydia* spp., and *Leptospira* spp. were included in routine screenings only from 2015 onwards. Light gray cells indicate no abortion outbreaks within that year.

The pathogens detected in abortion outbreaks in the 15 farms with the highest number of outbreaks during the study period are shown in Figure 3.

PRRSV was associated with abortion outbreaks in 14 out of 15 farms. In the breeding herd F6, PRRSV-related outbreaks were never recorded, and abortions were instead associated with *Chlamydia* spp. and PCV3. In six of the PRRSV-positive herds, the virus was detected in at least four out of 11 years, and in four herds it caused abortion outbreaks for five consecutive years. In breeding farm F4, the pathogen observed most frequently in association with abortions was *Chlamydia* spp. The first *Chlamydia* spp. outbreak in the farm occurred in 2016 and then annually between 2018 and 2021. PCV3 was detected in 13 farms and appeared to increase in frequency in recent years.

### 3.2. Factors Affecting Infection by Abortion-Inducing Pathogens

**Figure 4:**
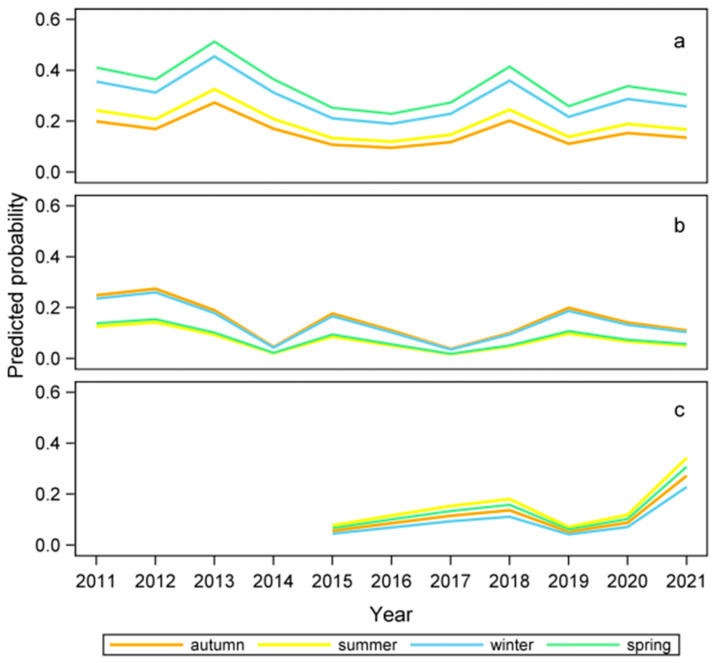
Probability by year and season for a pig abortion outbreak of being associated with PRRSV (**a**), PCV2 (**b**), and PCV3 (**c**). PCV3 was routinely included in the screening protocol only from 2015 onwards.

The probability of an outbreak being associated with PRRSV varied significantly by year (χ^2^_10_ = 18.4; *p* = 0.049) and season (χ^2^_3_ = 22.7; *p* < 0.0001). In detail, the prevalence of PRRSV-associated outbreaks peaked in 2013 and 2018 and was higher in winter and spring than in autumn and summer (Figure 4a). The density of pigs was not related to PRRSV presence (*p* > 0.05).

Regarding PCV2, we observed a slight reduction in its prevalence during the study period (χ^2^_10_ = 21.2; *p* = 0.023) and a strong seasonal variation in prevalence (χ^2^_3_ = 8.52; *p* = 0.039), with a higher probability of observing positive outbreaks in autumn and winter (Figure 4b). Conversely, the probability of PCV3 increased over time (χ^2^_6_ = 9.8; *p* = 0.021), with values in 2021 being significantly higher than those in 2015 (Figure 4c).

Finally, *Chlamydia* spp. infection varied by season (χ^2^_3_ = 9.9; *p* = 0.022), showing a significantly higher prevalence in autumn. It was inversely related to the density of pigs in the area (χ^2^_3_ = 4.0; *p* = 0.048), with high-density pig provinces showing lower prevalence.

Infection by the other examined pathogens (i.e., PPV and *Leptospira* spp.) was not significantly affected by any of the examined variables (all *p* > 0.05).

## 4. Discussion

We retrospectively investigated the main infectious agents detected in aborted fetuses submitted for analysis to our laboratory (IZSLER) over an 11-year period. These submissions were associated with over 800 abortion outbreaks that occurred in North Italian swine breeding herds. Overall, we observed a decreasing trend in abortion-related submissions during the study period. This reduction could stem from increased awareness among farmers and herd veterinarians of the importance of biosecurity measures and hygienic management of farrowing units, together with the increased application of complete sow vaccination protocols against the main abortigenic pathogens.

In almost half of the observed outbreaks (42%), we detected at least one abortion-inducing infectious agent. A similar detection rate was obtained in previous abortion studies carried out in Italy [22] and other countries [21,31]. Salogni and colleagues, who explored pathogens associated with abortions in the same geographical area from 2011 to 2013, obtained a slightly higher diagnosis rate than the one we obtained [22]. However, in contrast with that study, we investigated only two bacterial infections (*Chlamydia* spp. and *Leptospira* spp.), not taking into consideration opportunistic bacteria. Additionally, contrary to the previous study, in our case samples were considered positive for PCV2 only when the number of viral genomic copies was higher than 10^5^ [32].

PRRSV (genotype 1) was the main pathogen detected in association with abortion outbreaks during the study period (25% of abortion outbreaks), with some year-to-year variation in prevalence and a stronger association with outbreaks occurring during the colder season, in agreement with previous studies [33]. The annual prevalence of PRRSV in abortion cases remained relatively high during the study period, ranging from a minimum of 15.6% in 2016 to a maximum of 40.6% in 2013. The analysis of the subset of herds with the highest number of outbreaks confirmed that PRRSV is widespread in the region, as it was detected in almost all these farms and often in consecutive years. The high prevalence of PRRS reproductive outbreaks is a relevant factor with a significant economic impact on Italian breeding farm production. Moreover, the high mutation rate and recombination potential of PRRSV can lead to the emergence of highly pathogenic strains capable of inducing severe reproductive outbreaks or strains that evade vaccination-induced immune defenses [34,35]. For instance, in 2022, severe PRRS outbreaks characterized by high abortion rates, mortality in sows, and increased mortality in weaners and growers were reported in Spanish breeding farms [36]. The emergence and circulation of such strains highlight the need to improve the monitoring of abortion outbreaks in Italy by implementing whole-genome sequencing of PRRSV for the timely detection of highly pathogenic variants [37].

PCV3 was the second most common agent associated with abortion outbreaks in the present study (19.6% of abortion outbreaks), with a significant increase in prevalence in recent years, as reported by other authors [13]. As previously mentioned, there is still no consensus on the role of PCV3 as a causative agent of disease in swine, and its epidemiology, pathophysiology, and overall relevance as a cause of abortions in sows remain unclear [16]. The reported prevalence of PCV3 can also be highly variable depending on the diagnostic method and sample used [16]. In Italy, previous studies on symptomatic pigs reported values ranging from 12.5% [38] to 41% [39] and up to 69% when organ pools were tested [40]. Detection in asymptomatic pigs was relatively similar (approximately 30%) [39], while prevalences up to 60% have been reported in wild boars in the absence of clinical signs [41,42]. The proportion of abortion outbreaks associated with PCV3 (both mono- and coinfections) detected in the present study was lower than those reported in a previous Italian study [39].

In contrast to PCV3, the role of PCV2 as a causative agent of reproductive failure, particularly of late-term abortions and stillbirths, is well established [43]. The prevalence of PCV2-associated reproductive failure in field conditions is variable and has been described as ranging from 1 to 51% [17,22,44]. For instance, in Spain, PCV2 was detected by PCR in only one out of 100 outbreaks of reproductive failure in swine characterized by late-term abortions and premature farrowing [45]. In our study, we found a moderate prevalence (11.5% of abortion outbreaks), much lower than in a previous Italian study, which reported a 42.7% prevalence of PCV2-associated reproductive failures in the same geographical area [22]. However, this relevant difference might be because we used a different diagnostic threshold, namely, samples were considered positive only when values greater than 10^5^ PCV2 viral genomic copies/gram were detected. Values greater than 10^5^ PCV2 viral genomic copies/gram have been detected in tissues of fetuses with myocarditis, and values greater than 10^7^ PCV2 DNA copies/gram have been described as strongly indicative of PCVAD [32,46]. Indeed, if we did not consider the viral load, the number of samples positive for PCV2 in our dataset would be three times as high. Additionally, we observed a slow but significant reduction in PCV2-associated outbreaks during the 11-year study period. Therefore, we cannot exclude the possibility that the occurrence of PCV2-associated reproductive failures in 2009–2013 [22] was actually higher. The decision to use different positivity thresholds for PCV2 and PCV3 is based on the current understanding of the epidemiology of these two viruses in their swine host. For PCV2, specific diagnostic criteria have been established, including the quantification of viral genome copies using quantitative PCR protocols, which helps to differentiate between subclinical infections and those associated with systemic or reproductive disease [29]. In contrast, although a similar diagnostic approach has been proposed [13,30], no specific criteria are available for PCV3 to date.

The decline of PCV2 during the last decade could be explained by the high vaccination rate in the Italian pig industry, especially in breeders. Vaccination of sows is highly effective in conferring passive immunity to piglets against PCV2, and since commercial vaccines became available in 2006, their use has rapidly increased worldwide [47].

Several *Chlamydia* spp. can infect pigs, with the two most common being *C. suis* and *C. abortus* [18]. Serological surveys in intensive pig farms located in northern Italy found a *Chlamydia* spp. seroprevalence above 60% [48]. Nevertheless, in the present study, *Chlamydia* spp. was associated with abortion outbreaks only sporadically (6% of abortion outbreaks), indicating a low or occasional role as a reproductive pathogen. A notable exception was a single farm where multiple abortion outbreaks associated with *Chlamydia* spp. were observed for several consecutive years, suggesting some specific management issues. Infection in our sample varied by season, showing a significantly higher prevalence in autumn. Chlamydiosis in humans and most other species is not considered seasonal, and few data exist relative to *Chlamydia* spp. seasonality in pigs. For instance, a recent meta-analysis by Sheng and colleagues on *Chlamydia* spp. infection in pigs in China did not find any specific association with seasons but rather an influence of climatic parameters: higher prevalences were observed in warm, humid climates characterized by heavy rainfall [49]. Contrary to our predictions, we also found a negative association between *Chlamydia* spp. infection in fetuses and the density of pigs in the area. A tentative explanation for this unexpected result might lay in the density variable actually reflecting differences in herd management between geographic areas. Indeed, high-density pig provinces are characterized almost exclusively by intensive farming, with large, often multisite operations where pig flows are usually more controlled and levels of biosecurity are higher. For instance, while Hoffmann and colleagues reported no association between *Chlamydia* spp. infection and the absolute number of pigs in a herd, they found a higher infection risk associated with increasing numbers of pigs of different origins [50]. Detailed farm management data would be needed to better disclose this negative association.

The detection of PPV and *Leptospira* spp. in our sample was even more sporadic than *Chlamydia* spp. (4% and 2.6% of abortion outbreaks, respectively). PPV infection has been widespread in the Italian pig population since the 1980s [51]. For this reason, vaccination of breeders, and particularly of gilts, before the first insemination is widely practiced. Inactivated vaccines against PPV, as well as natural infection, are known to induce long-lasting immunity and usually protect fetuses from disease even from heterologous strains [52,53], which may explain the low detection of PPV in the examined outbreaks. *Leptospira* spp. infection was a widespread cause of clinical disease in the swine industry, but with a shift to indoor production, its circulation appears to have decreased, possibly due to reduced contact with wildlife reservoirs [19]. Recent surveys of Italian slaughtered pigs revealed a seroprevalence between 16.4% in southern Italy [54] and 13% in northern Italian farms [55].

Most of the outbreaks were characterized by late-term abortions. Similarly, in a previous Italian survey, 71% of analyzed abortion cases occurred in the second half of pregnancy [22]. This could be justified by a greater difficulty in finding small fetuses aborted in the first part of gestation in the sow’s bedding or by the possible occurrence of embryonic resorption, which could be mistaken as a fertility problem. As expected, PRRSV infection was strongly associated with late-term abortions. Indeed, although fetuses of all ages are susceptible to PRRSV infection, transmission by the mother to the fetuses and virus replication in placenta and fetal tissues are more efficient in the last third of gestation [8]. Conversely, *Chlamydia* spp. was mainly associated with early abortions. Clinical presentation of genital tract infection by *Chlamydiaceae* greatly varies based on the age of gestation. Early embryonic death and increased rates of return to oestrus have been frequently described, but abortions, stillbirth, and increased perinatal and neonatal mortality can also be observed [18,56].

Coinfections by different pathogens in abortion outbreaks in swine farms are common, and among them, consistent with our findings, PRRSV + PCV is usually the most frequently diagnosed combination [57,58,59]. This trend is also visible by observing the temporal pattern in our subset of 15 farms: only in one farm, PRRSV and PCV were not involved in abortive outbreaks during the study period. Coinfection with PCV2 and PRRSV in aborted fetuses and stillborn piglets was previously reported, and synergistic relationships between the two infectious agents enhancing damage to fetal structures or to the maternal–fetal interface have been speculated [59]. A high prevalence of coinfections in cases of Stillbirth, Mummification, Embryonic Death, and Infertility (SMEDI) syndrome was described in Germany [60]. The authors reported an increased probability of detecting *Leptospira* spp. in association with PCV2, suggested a possible inhibitory effect of PCV3 on the pathogenesis of PCV2 infection in fetuses, and described an enhanced negative effect concerning the development of a fetus in the case of coinfection with PCV2 and PPV1 [60]. Disclosing coinfection dynamics in multifactorial syndromes is a challenging task. Histopathology and immunohistochemistry performed on fetal samples could better clarify the role of each detected microorganism, providing a wider understanding of such dynamics [59].

The application of a standardized and comprehensive diagnostic protocol, including targeted analyses for the most epidemiologically relevant pathogens, is a valuable tool to address abortion outbreaks and implement the most appropriate management strategies, limiting further economic losses. In this regard, an important limitation of the present study concerns its retrospective nature. Data collection was based on sampling conducted voluntarily by herd veterinarians over the years, who sometimes specifically requested a partial diagnostic protocol from the laboratory that did not include all the abortigenic agents considered in our study. This may have additionally influenced the observed etiological detection rate. Furthermore, little to no information about herd management (e.g., vaccination strategies of sows and gilts) and health status, in particular on abortion rates, was reported on the submission documents, which could have contributed to a better interpretation of our results. Success in controlling and preventing abortion outbreaks in sows requires indeed a holistic approach that includes not only laboratory analysis but also an in-depth evaluation of the herd’s health status and management.

Nevertheless, the present study represents the first comprehensive survey on abortigenic pathogens circulating in the main Italian swine production area over a long period of time, and we believe that our results provide useful knowledge towards a better optimization of sow health management and greater rationalization of on-farm prevention and control measures.

## 5. Conclusions

Reproductive failure in sows, especially abortion, is a significant factor affecting pig herd profitability. Considering the complex multifactorial nature of abortion outbreaks, our results underline the importance of adopting a consistent and standardized sampling procedure and a complete diagnostic protocol that minimizes the inconclusive diagnosis rate. Detailed knowledge of pathogen circulation is indeed essential to adopt targeted biosecurity measures and to plan effective vaccination strategies, limiting the economic impact of reproductive disease. This is a realistic goal, considering the decreasing trend in abortion outbreaks observed during our study period. Using the present data as a starting point, future studies may expand our understanding of the role of pathogens in abortion outbreaks by applying appropriate diagnostic protocols by farm risk analysis and with the use of more informative diagnostic tools (e.g., next-generation sequencing technologies), potentially leading to disclosure of new causative associations.

## Figures and Tables

**Table 1:** Diagnostic methods used to investigate abortion-inducing pathogens on pig fetuses.

Pathogen	Period	Material	Method	References	Positivity Cut-Off
PCV2	2011–2021	pooled organs (heart, lung, and liver)	quantitative real-time PCR	[24]	LoQ 10^5^ copies/mL of homogenate
PCV3	2015–2021	pooled organs (heart, lung, and liver)	real-time PCR	primers described in [15]; probe described in Table S1	Cq 38
PPV	2011–2014	pooled organs (heart, lung, and liver)	PCR	[25]	
PPV	2015–2021	pooled organs (heart, lung, and liver)	real-time PCR	[26]	Cq 38
PRRSV	2011–2021	pooled organs (heart, lung, and liver)	real-time RT-PCR	Virotype PRRSV RT-PCR Kit, Indical^®^, Leipzig, Germany	Cq 37
*Chlamydia* spp.	2015–2021	pooled organs (heart, lung, and liver)	real-time PCR	[27]	Cq 38
*Leptospira* spp.	2015–2021	kidney tissue	real-time PCR	[28]	Cq 40

**Table 2:** Prevalence and 95% Confidence Interval (95% CI) of selected pathogens detected in pig abortion outbreaks submitted for analysis from 2011 to 2021. In 480 out of 829 abortion outbreaks (58%), none of the tested pathogens were detected.

Pathogens	No. of Outbreaks Tested	No. of Positive Outbreaks	Prevalence (%)	95% CI (%)
**PCV2**	790	91	11.5	9.3–13.7
**PCV3 ^a^**	438	86	19.6	15.9–23.4
**PPV**	680	27	4.0	2.5–5.4
**PRRSV**	817	204	25.0	22.0–27.9
***Chlamydia*** **spp. ^a^**	537	30	5.6	3.6–7.5
***Leptospira*** **spp. ^a^**	490	13	2.6	1.2–4.1

^a^ tested from 2015 onwards.

**Table 3:** Combinations of pathogens detected in pig abortion outbreaks (*n* = 87) showing multiple coinfections.

Pathogens	No. of Pathogens	No. of Positive Outbreaks	%
PRRSV + PCV2	2	28	32.2
PRRSV + PCV3	2	20	23.0
PRRSV + *Chlamydia* spp.	2	6	6.9
PCV2 + PCV3	2	4	4.6
PCV3 + *Chlamydia* spp.	2	4	4.6
PRRSV + PPV	2	4	4.6
PRRSV + PCV2 + PPV	3	4	4.6
PCV3 + *Leptospira* spp.	2	3	3.4
PRRSV + PCV2 + PCV3	3	3	3.4
PCV2 + PPV	2	2	2.3
PCV2 + PCV3 + *Chlamydia* spp.	3	2	2.3
PCV2 + *Leptospira* spp.	2	1	1.1
PCV2 + PCV3 + PPV	3	1	1.1
PCV3 + *Leptospira* spp. + *Chlamydia* spp.	3	1	1.1
PCV3 + PPV	2	1	1.1
PRRSV + *Leptospira* spp.	2	1	1.1
PRRS + PPV + *Leptospira* spp.	3	1	1.1
PRRS + PCV3 + PPV	3	1	1.1

## Data Availability

The data presented in this study are available on request from the corresponding author. The data are not publicly available due to privacy restrictions.

## Supplementary Materials

The following supporting information can be downloaded at: https://www.mdpi.com/article/10.3390/vetsci11100496/s1. Table S1: Sequences of the oligonucleotides used for detecting PCV3.
