# Supplementary material for: Infectious Agents Associated with Abortion Outbreaks in Italian Pig Farms from 2011 to 2021
Source: Vet Sci. 2024 Oct 12;11(10):496. doi: 10.3390/vetsci11100496 (PMC11512215; doi:10.3390/vetsci11100496)
Supplement: Supplementary file 1 [file vetsci-11-00496-s001.zip › vetsci-3204857-supplementary.pdf]

Veterinary Sciences

Infectious agents associated with abortion outbreaks in Italian pig farms from 2011 to 2021

Anna Donneschi[1†], Matteo Recchia[1†], Claudia Romeo[1,2*], Paolo Pozzi[3], Cristian Salogni[1], Antonio Marco Maisano[1], Giovanni Santucci[1], Federico Scali[1], Silvia Faccini[1], Maria Beatrice Boniotti[1], Mario D'Incau[1], Dominiek Maes[4], Giovanni Loris Alborali[1]

[1] Istituto Zooprofilattico Sperimentale della Lombardia e dell'Emilia Romagna – IZSLER, Brescia, Italy; anna.donneschi@gmail.com (A.D.); matteo.recchia@izsler.it (M.R.); cristian.salogni@izsler.it (C.S.); antoniomarco.maisano@izsler.it (A.M.M.); giovanni.santucci@izsler.it (G.S.); federico.scali@izsler.it (F.S.); silvia.faccini@izsler.it (S.F.); mariabeatrice.boniotti@izsler.it (M.B.B.); mario.dincau@izsler.it (M.D.I.); giovanni.alborali@izsler.it (G.L.A.)
[2] Center for Evolutionary Hologenomics—Globe Institute, University of Copenhagen, Øster Farimagsgade 5, 1353 Copenhagen, Denmark
[3] Dipartimento di Scienze Veterinarie, Università degli Studi di Torino, 10095 Grugliasco (TO), Italy; paolo.pozzi.s@gmail.com (P.P.)
[4] Department of Internal Medicine, Reproduction and Population Medicine, Faculty of Veterinary Medicine, Ghent University, Merelbeke, Belgium; Dominiek.Maes@UGent.be (D.M.)
[†] These authors contributed equally
* Correspondence: claudiarosa.romeo@izsler.it (C.R.)

Table S1 - Sequences of the oligonucleotides used for detecting PCV3

	Sequence 5' – 3'	fragment size	References
PCV3_F	AGTGCTCCCCATTGAACG		Palinski et al. 2017
PCV3_R	ACACAGCCGTTACTTCAC	112 bp	Palinski et al. 2017
PCV3-probe	FAM-AAGAAGAGGCTTTGTCCTGGGTGA-BHQ1		This study

References

Palinski R, Piñeyro P, Shang P, Yuan F, Guo R, Fang Y, Byers E, Hause BM. A Novel Porcine Circovirus Distantly Related to Known Circoviruses Is Associated with Porcine Dermatitis and Nephropathy Syndrome and Reproductive Failure. J Virol. 2017;91(1):e01879-16.
